# Women's lived experience of incarceration in Kobo Prison Center, Ethiopia: implications for social work practice

**DOI:** 10.3389/fgwh.2025.1561108

**Published:** 2025-09-29

**Authors:** Sindew Asmare, Tesfa Anmut, Siyoum Mekonnen

**Affiliations:** 1 Department of Sociology, Woldia University, Woldiya, Ethiopia; 2 Department of Social Work, Wollo University, Dessie, Ethiopia

**Keywords:** women prisoners, Ethiopia, social work, empowerment, correctional services, gender-responsive programs, qualitative phenomenology

## Abstract

**Introduction:**

Ethiopian women in prison face unique and exacerbated issues that are not adequately addressed by the criminal justice system, especially when it comes to gender responsive social work services. This study uses a qualitative phenomenological design to investigate the lived experiences of women who are incarcerated in the Amhara region at Kobo Prison. The study focuses on the ways in which social work interventions affect the psychosocial well-being and empowerment of these women.

**Methods:**

Seven participants were specifically chosen from among the roughly 70 female inmates housed at Kobo Prison to offer a range of viewpoints on the experiences of incarceration. Owing to COVID-19 limitations, open-ended written questionnaire that participants filled out in private were used to collect data, guaranteeing confidentiality. An inductive thematic approach was used to manually analyze the responses.

**Results:**

Findings indicate that incarcerated women face significant barriers including inadequate mental health support, lack of childcare provisions, and insufficient protection from gender based violence, and limited access to vocational and educational opportunities. The informal, underfunded, and frequently gender-insensitive social work services provided in prisons increase women's vulnerabilities and impede their rehabilitation. Notwithstanding these obstacles, counseling services and skill building exercises turned out to be crucial facilitators of empowerment and resilience.

**Discussion:**

The study emphasizes how important it is to establish gender responsive social work programs that are specifically designed to meet the needs of Ethiopian women who are incarcerated. By putting women's voices front and center, the study adds important knowledge to the little studied field of correctional social work in low-income settings and offers data to support program development and policy changes. In the end, the results support comprehensive, gender-sensitive strategies to enhance the wellbeing and social reintegration of women who are incarcerated.

## 1 Introduction

### 1.1 Women in prison globally

Approximately 700,000 women are⁠ incarcerated worldwide, many⁠ facing unique and complex needs related to health, caregiving, and social reintegration (1). Globally, women in prisons are⁠ more⁠ likely than men to experience mental health issues, trauma histories, and gender based violence, all of which require tailored correctional programs (2). Despite these well documented challenges, prison systems and rehabilitation initiatives often remain gender-neutral or male-focused, frequently neglecting the specific needs of incarcerated women (3).

### 1.2 Women in prison in Ethiopia

In Ethiopia, approximately 5,000 women are held across federal and regional prisons (4). A significant number are mothers, with children either living with them in prison or left behind in the community. Many women are incarcerated for economic crimes such as petty theft or survival related offenses (5, 6). These women often enter prison with preexisting vulnerabilities including poverty, limited⁠ formal education, trauma from gender-based violence, and barriers to stable employment (7–10). Prison conditions frequently exacerbate these challenges through separation from children, restricted access to mental health and⁠ vocational services, and social marginalization (11, 12). Despite the high prevalence of psychological distress, counseling and psychosocial supports remain scarce within Ethiopian prisons (13, 14).

### 1.3 Prison programs addressing women's needs

Internationally, some prison systems have⁠ has begun implementing gender-⁠sensitive programs focusing on menstrual hygiene management, mental health support, parenting, and protection from gender-based violence (15, 16). These programs aim to reduce recidivism by fostering empowerment, self⁠-efficacy, and successful social reintegration. However, such gender-specific initiatives remain limited or absent in many low-⁠income countries, including Ethiopia, due to systemic constraints and resource limitations (17, 18). The COVID-19 pandemic has further disrupted these services by limiting external visits and program delivery (19, 20).

### 1.4 Social work in correctional settings

Social work is a professional practice rooted in human rights, social justice, and collective well-being, with a core aim to⁠ empowers individuals, families, and communities to improve social functioning and quality of life through problem-solving, resource access, and advocacy (21). Within correctional settings, social work plays a vital rehabilitative role by addressing psychosocial challenges, promoting self-efficacy, supporting family reunification and facilitating⁠ reintegration into⁠ society (16, 22).

### 1.5 Social work in Ethiopian prisons

In Ethiopia, social work within prisons remains underdeveloped and largely informal. Correctional social work is not yet fully institutionalized as a profession, with services often⁠ delivered by personnel lacking specialized training in social work or rehabilitation. These may include volunteers or staff assigned general welfare roles rather than professional social workers (18, 23). Collaborations with civil society organizations and NGOs exist but is inconsistent and largely⁠ dependent on external funding and temporary programs. Importantly, Ethiopian prison social work lacks comprehensive gender-sensitive programming tailored to the distinct needs of incarcerated women. Critical issues⁠ such as⁠ menstrual hygiene management, mental health support, childcare arrangements, and protection from gender⁠-based violence are inadequately addressed. These gaps worsen women's vulnerabilities and hinder their rehabilitation and empowerment (13, 15, 17). The COVID-19 pandemic further strained these services by restricting external visits and limiting program delivery (19, 20).

### 1.6 Women's⁠ experiences in Ethiopian prisons

Women incarcerated in Ethiopia often face compounded vulnerabilities including poverty, limited education, trauma, and unemployment barriers (5, 6). Correctional conditions tend to amplify these difficulties, especially through separation from children, social isolation; and limited access to mental health and vocational training programs (7–9, 16). Psychological distress is common; yet counseling and psychosocial support remain minimal (13, 14). Maternal incarceration disrupts family care arrangements, placing children at increased risk of neglect or institutionalization (11, 12).

### 1.7 Research gap and study objective

Despite the critical role of gender-responsive correctional social work, limited knowledge exists on how this programs⁠ impact incarcerated women in Ethiopia, particularly regarding⁠ their empowerment and rehabilitation. This study fills this gap by employing a qualitative phenomenological approach to explore women's lived experiences of incarceration and⁠ their interactions with social work services in an Ethiopian federal prison. Grounded in empowerment theory—which emphasizes autonomy, self-⁠efficacy, resilience, and social reintegration capacity (24–26)—the research aims to examine how existing programs support or hinder empowerment processes. The ultimate goal is to inform the development of gender responsive, equitable, and institutionalized correctional social work practices tailored to the unique psychosocial needs of incarcerated women.

## 2 Literature

### 2.1 Empowerment framework

Empowerment, in the context of this study, refers to⁠ the process through which incarcerated women gain the ability and opportunity to exercise agency, achieve autonomy over their lives, and overcome structural and systemic barriers that constrain their social, economic, and psychological well-being both within and beyond correctional settings. This concept recognizes empowerment as multidimensional, encompassing individual capacities⁠, social relationships,⁠ and institutional factors that collectively influence⁠ control over⁠ one's circumstances (26–28).

Drawing on (28) psychological⁠ empowerment theory, empowerment is conceptualized as⁠ a dynamic and interactive process comprising⁠ three components: intrapersonal, interactional, and behavioral. The intrapersonal component refers to individuals' perceptions of control and self-efficacy—the belief in one's ability to influence outcomes⁠. The interactional component involves critical awareness and understanding of social and structural contexts, including the identification of barriers and resources needed to exert control. The behavioral component reflects actions taken to exert influence, such⁠ as seeking⁠ resources, participating in programs, or advocating⁠ for needs. Within correctional settings, these components highlight that empowerment involves⁠ not only personal confidence but also navigating institutional constraints and opportunities (14, 28).

Similarly, Kabeer (27) offers a complementary framework emphasizing empowerment as the process⁠ by which those denied the ability to⁠ make strategic life choices acquire that ability. Empowerment requires three interrelated elements: resources (material, human, and social), ⁠ agency (the ability to define goals and act upon them), and achievements (the outcomes of agency). This framework underscores that empowerment is not only an individual attribute but also deeply influenced by social structures, power⁠ relations,⁠ and resource access. For incarcerated women, deprivation of liberty and social stigma exacerbate disempowerment, limiting access to educational, vocational, and psychological support necessary for meaningful reintegration ( ⁠^26^).

Linking empowerment to autonomy, self-efficacy⁠, and decision-making, empowerment is framed as a critical outcome of effective social work and rehabilitation⁠ services. Autonomy refers to the capacity to make informed and un⁠co⁠erced decisions; self-efficacy reflects⁠ confidence in one's ability to act; decision-making denotes⁠ the ability to translate intentions into actions. These elements are essential for incarcerated women who face compounded vulnerabilities including gender discrimination, social exclusion, and trauma histories (15, 29, 30). Data themes emerging from this study reflect barriers to empowerment such as limited awareness of social work services, gender-insensitive programming, inadequate vocational and educational opportunities, and stigmatizing attitudes within the correctional system, conversely, enablers include⁠⁠ access to counseling, skill building programs, and empathetic social work interventions fostering resilience and self-worth ( ⁠^22^, ^26^).

By interpreting the lived experiences of incarcerated women through this empowerment lens, the study reveals how correctional social⁠ work practices can hinder or facilitate agency and social reintegration. This underscores⁠ the need for gender responsive, holistic approaches addressing individual psychosocial needs alongside structural inequalities embedded in prison policies and practices (19, 20).

### 2.2 Legal/policy context

Ethiopia has developed policies aimed at reforming its prison and criminal justice system, including the National Criminal Justice Policy (31) and the Prison Reform Strategy (32), which emphasizes rehabilitation and reintegration. However, these policies provide limited gender specific provisions addressing the distinct needs of incarcerated women. Critical areas such as menstrual hygiene management, mental health services, childcare support, and protection from gender-based violence are inadequately covered or absent (18, 33, 34), this policy gap results in insufficient structural support for women's unique rehabilitation needs, complicating efforts to foster empowerment⁠ and social reintegration.

For social work practice, these shortcomings highlight the necessity of advocacy for gender responsive policy reform and implementations⁠ of specialized services tailored to women's vulnerabilities within correctional settings. Consequently, social workers must navigate these gaps to provide holistic care addressing both individual psychosocial needs and systemic inequalities affecting incarcerated women and their families (13, 17, 34).

## 3 Methodology

### 3.1 Research design and philosophical stance

This study employed a qualitative phenomenological design to explore the lived experiences of women incarcerated at Kobo Prison in the Amhara region. The aim was⁠ to understand participants’ subjective experiences of prison life and social work services from their own perspectives. The study was grounded in constructivist epistemology, which assumes that knowledge is co-constructed through social interaction and individual meaning-making. To enhance rigor, researcher biases were consciously bracketed during data analysis to maintain focus on participants' narratives.

### 3.2 Sampling and sample size justification

Participants were purposively sampled to capture a⁠ diverse range of perspectives on the lived experiences of incarcerated women at Kobo Prison⁠, which houses approximately 300 inmates, including around 30 women. This sampling approach prioritizes depth and variation over statistical generalizability, consistent with qualitative phenomenological research principles (35, 36). All 30 eligible women meeting the criteria of current incarceration at Kobo Prison and the ability to⁠ provide written responses were invited to participate through information distributed by prison administrator's acting⁠ as gatekeepers.

Seventeen women voluntarily completed the initial written questionnaires, providing a preliminary data set. From these, 7 participants were purposively selected for more detailed⁠ phenomenological analysis. Selection focused on maximizing variation in demographic and experiential characteristics—such as age, conviction status (pre-trial vs. sentenced), and length of incarceration—to reflect the broader population's diversity. The decision to focus on 7 participants aligns with standard qualitative guidelines recommending smaller samples in phenomenological studies to facilitate deep, nuanced exploration of participants’ lived experiences. For instance, Creswell (35) suggests phenomenological samples typically range between 5 and 25 participants, while Guest et al. (2⁠006) emphasize that saturation—the point at which no new themes emerge—often occurs within 6–12 interviews.

Additionally, the 7⁠ selected narratives demonstrated sufficient richness and depth to support a thorough phenomenological interpretation. This sample size balances the need for comprehensive data analysis with practical constraints, such as resource limitations and ethical considerations related to working within a correctional setting. The written responses reviewed at this stage consisted of detailed, open-ended reflections elicited by semi-structured questionnaires exploring participants' prison life, personal histories, engagement with social work services, and future aspirations. This approach ensured data quality appropriate for the study's phenomenological⁠ aims.

### 3.3 Data collection

Due to COVID-19 restrictions, traditional face-to-face interviews were not⁠ feasible. Instead, data were collected through open-ended written questionnaires completed by participants in private rooms within Kobo Prison, with no researchers or prison staff present during⁠ completion. This method was⁠ chosen to ensure participant confidentiality, reduce social desirability bias, and align with institutional health guidelines. The questionnaire consisted of 10 open-ended prompts designed to elicit rich, narrative⁠ responses related to the research objective—specifically, to explore women's experiences of incarceration and the perceived role of social work in promoting empowerment and rehabilitation.

The instrument was structured around three temporal domains—before, during, and after incarceration—reflecting a holistic view of empowerment as a process influenced by personal history, institutional context, and future orientation. Key prompts included:

1. **Before Incarceration:**
  - “Can you describe the events or circumstances in your life that led to your incarceration?”
  - “What types of support (if any) did you receive from your family, community, or institutions before imprisonment?”
  **Rationale**: These questions help contextualize participants' vulnerabilities and identify gaps in pre-incarceration support that may relate to social work interventions.
2. **During Incarceration:**
  - “What is a typical day like for you in prison?”
  - “Have you had any contact with social workers during your incarceration? If so, please describe the experience.”
  - “What kinds of support services are available to you here (e.g., counseling, rehabilitation, legal aid)?”
  - “What are the biggest challenges you face emotionally or psychologically?”
  **Rationale**: These questions directly relate to the study's aim of assessing the presence and perceived impact of social work interventions and other supportive services in the correctional setting.
3. **Looking Ahead:**
  - “How do you feel about your future after release?”
  - “What support or preparation do you think would help you reintegrate into your community?” ⁠
  - “What role do you think social workers or similar pro⁠fe⁠ss⁠ionals should play in your⁠ life after prison?”
  **Rationale**: These prompts assess future expectations and highlight how participants view empowerment and support, especially⁠ in terms of reentry, which is a core concern of social work practice. Participation in the study was fully voluntary. No material or financial incentives were offered. The responses collected⁠ were later coded and analyzed thematically to identify key themes related to empowerment, institutional experience, and the role of social work.

### 3.4 Data analysis

Data analysis was conducted manually by the primary researcher following an inductive thematic analysis approach adapted from Braun and Clarke (37). This process involved repeated readings of responses to become familiar with the data,⁠ coding meaningful segments without predetermined categories, and grouping codes into emergent themes reflecting shared⁠ patterns and unique experiences. Themes were reviewed for coherence and relevance, then defined and named to represent participants' perspectives accurately. Original responses, written in Amharic, were translated into English with cross-checking to ensure accuracy. Although qualitative analysis software and a second coder was not available, peer debriefing sessions were held to discuss⁠ emerging themes and minimize researcher bias.

### 3.5 Researcher reflexivity and bias control

To ensure rigor and minimize bias, the study employed multiple strategies. Researcher triangulation involved three researchers independently coding subsets of data, followed by team discussions to reconcile discrepancies. This process yielded an interceder agreement rate of approximately 85%, enhancing reliability. Bracketing techniques were applied by documenting researchers'⁠ preconceptions through reflective memoing throughout data collection and analysis, helping maintain focus on participants' authentic narratives. Regular peer debriefing sessions further supported critical examination of interpretations and challenged potential biases⁠. These combined approaches contributed to the trustworthiness of findings by ensuring themes reflected the⁠ lived experiences of incarcerated women rather than researcher assumptions.⁠

### 3.6 Ethical considerations

Ethical approval was granted by the institutional review board overseeing the research. Written informed consent was obtained from all participants, who were assured of their voluntary involvement and confidentiality, while researchers were aware of participants' identities to facilitate data⁠ collection and follow-up, personal information was securely stored and anonym in all reporting. No incentives were provided. The study prioritized confidentiality over anonymity to maintain trust while protecting participant privacy. To enhance trustworthiness, the⁠ study applied several strategies:

- **Credibility**: Through rich descriptive data, participant verification of select⁠ excerpts, and researcher reflexivity.
- **Transferability**: By providing detailed descriptions of the context and participant profiles.
- **Dependability**: Via clear documentation of the coding and analysis processes.
- **Conformability**: Through the use of a subjectivity statement and systematic efforts to bracket researcher biases.

### 3.7 Limitations

This study's findings derive from a small, purposive sample within a single prison, limiting the generalizability to other prisons in Ethiopia or beyond. The use of written questionnaires, while necessary under pandemic restrictions, restricted the depth and spontaneity of participant responses, especially concerning sensitive topics. Nonetheless, the research provides valuable insights into incarcerated women's experiences and the role of social work in Ethiopian correctional settings, establishing a foundation for future, more comprehensive studies.

### 3.8 Linking data collection to research objectives

To ensure clarity on how the⁠ data collection aligns with the study's aims, each question in the semi-structured questionnaire was designed to explore key aspects of women's incarceration experiences that relate directly to empowerment and the role of social work interventions. The questions were grouped to capture a holistic picture, covering life before incarceration, experiences during imprisonment, and expectations for the future, specifically:

✓ Questions about life before prison aimed to understand the social, economic, and familial contexts shaping women's vulnerabilities and pathways to incarceration. This background information provides essential context for understanding empowerment needs and barriers.
✓ Questions about daily life in prison and interactions with social work services were designed to assess perceptions of support, access to resources, and empowerment opportunities facilitated by social workers. For example, inquiries about social work programs, counseling, or rehabilitation initiatives helped evaluate the role of social work in addressing psychosocial challenges and promoting agency.
✓Questions about future aspirations and post⁠-release plans explored how incarceration and social work interventions influenced women's sense of hope, self-efficacy, and empowerment moving forward.

## 4 Findings

### 4.1 Overview and theoretical lens

This section presents key findings from written responses provided by seven incarcerated women at Kobo Prison. Drawing on Zimmerman's (28) psychological empowerment framework, the analysis is⁠ organized into three major dimensions of empowerment:

- Intrapersonal (self-concept, confidence, perceived control),
- Interactional (critical awareness of systems, ability to navigate⁠ institutions), ⁠ and
- Behavioral (action-taking, participation in change, future orientation).

Each thematic category illustrates the extent to which participant's experience—or is denied—empowerment within the correctional context, particularly in relation to social work services.

### 4.2 Participant demographics and background

The study involved seven incarcerated women at Kobo Prison in the Amhara⁠ region, Ethiopia. Participants ranged in age from 21 to 35 years, with varying marital statuses, parental roles, and sentences (3–30 years). Table 1 summarizes their socio-demographic profiles, including pseudonyms used throughout this study to humanize their narratives.

**Table 1:** Socio-demographic characteristics of women incarcerated in Kobo Prison Center, Ethiopia.

Pseudonym	Age	Marital status	Children	Sentence length	Time served	Previous occupation
Hana	35	Married	No	20 years	6 years	Government employee
Selam	35	Single	No	30 years	15 years	Student
Mahi	34	Married	Yes	3 years	8 months	Employed (unspecified)
Almaz	27	Single	No	5 years	2.5 years	Legal expert, govt worker
Genet	30	Married	Yes (2)	14 years	2 years	Government official
Tigist	21	Single	No	4.5 years	2 years	Student
Saba	23	Single	No	8 years	5 years	Student

### 4.3 Thematic analysis

Thematic analysis of participants' responses revealed three primary themes related to empowerment and social work practices:

- Theme 1: Access to Psychosocial Support
- Theme 2: Challenges in Prison Environment
- Theme 3: Hope and Rehabilitation To better illustrate the themes,

Table 2 summarizes the themes, subthemes, illustrative participant quotes, and interpretations.

**Table 2:** Themes and subthemes identified from women's lived experiences of incarceration.

Theme	Subtheme	Illustrative quote	Interpretation
Access to psychosocial support	Availability of counseling	“The social worker listens to me and gives hope.”	Social work contributes positively to empowerment by providing emotional support.
Challenges in prison environment	Lack of resources	“There are not enough books or activities here.”	Prison conditions limit the effectiveness of empowerment strategies.
Hope and rehabilitation	Future orientation	“I feel I can start anew when I get out.”	Empowerment includes fostering hope and motivation for change.

(Figure 1 presents a conceptual model showing how these themes interconnect to support empowerment).

**Figure 1:**
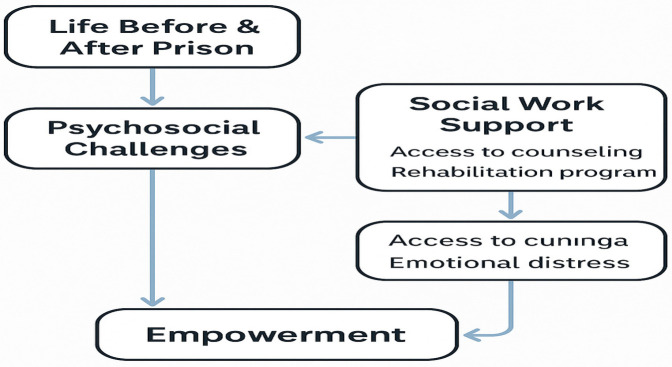
Conceptual model illustrating how key themes interconnect to support the empowerment of incarcerated women.

### 4.4 Life before incarceration: social identity and system trust

#### 4.4.1 Subtheme: identity and aspirations

Participants' reflections on their pre incarceration lives were filled with nostalgia and a clear contrast between⁠ their former and current selves. Most described themselves as ambitious, self-reliant, and socially responsible individuals who had meaningful roles in their communities.

“I used to be a hardworking woman, always eager to help others.”—Mahi

“I was always busy—working and going to school⁠. I didn’t even have time for family.”—Almaz

These reflections suggest that incarceration had⁠ not only interrupted their external lives but had also fractured their internal⁠ self-concept. A recurring theme was the sense of loss of identity, particularly regarding⁠ their⁠ role in society and⁠ the potential they once believed they had.

#### 4.4.2 Subtheme: trust in legal and social institutions

Before imprisonment, several participants held positive views about the legal system, human rights, and the importance of dialogue. However, incarceration led to a significant erosion of trust in social institutions.

“I used to believe in negotiation and rights. Now I don’t trust my country or the legal system.”—Almaz

“Prison is like university. I’ve learned a lot about social life… how to manage relationships.”—Saba

This shift illustrates how incarceration can alter not just personal trajectories but fundamental worldviews, including views on fairness, justice, and trust. Participants⁠ recognized broader patterns of social exclusion, gender inequality and institutional neglect.

### 4.5 Lived experience of incarceration

#### 4.5.1 Subtheme: psychological and emotional impact

Nearly all participants' reported mental health strain, hopelessness⁠, and profound emotional isolation.⁠

“Being locked in prison for doing a good deed—it all seems like a dream.”—Mahi

“I have lost hope⁠… I don’t think about anything except how to get out⁠.”—Almaz⁠

COVID-19 amplified these effects by eliminating family visits and other supports.

#### 4.5.2 Subtheme: gendered experience of prison

Participants highlighted the unique burdens related to motherhood, fertility, and caregiving, emphasizing gender-specific injustices such as limited reproductive health access and erasure of maternal identity.

“Every woman wants to be a mother… but she might never get the chance.”—Selam

### 4.6 Encounter with social work and support services

#### 4.6.1 Subtheme: limited access and understanding

Only four participants reported any awareness of counseling or social work, but services were vague,⁠ informal, and inconsistent, with no clear presence of trained professionals.

“Counseling is available Monday to Friday. They guide us on behavior change.”—Selam

#### 4.6.2 Subtheme: dissatisfaction and critique

Participants were dissatisfied with the lack of structured social work services, poor communication access, and insufficient rehabilitation programming.

“There are correctional centers—but who is correcting who?”—Hana

“They think simple things like phone calls are luxuries.”—Almaz

#### 4.6.3 Subtheme: vision for reform

Participants offered practical suggestions including gender-sensitive programming, vocational training, education, psychological counseling, and better-trained staff.

 “Training should reflect our personalities and backgrounds.” —⁠ Genet

“The staffs themselves need training… I doubt they understand rehabilitation.”—Almaz

### 4.7 Imagining the future: hope, advocacy, and reintegration

#### 4.7.1 Subtheme: resilience and self-development

Despite trauma, participants expressed determination to pursue education, advocacy, and personal growth post-release.

“Maybe I’ll become a better advocate for rights… join an opposition party.”—Almaz

“You improve. You learn. You move on.”⁠—Genet

#### 4.7.2 Implications for social work practice

This broader framing allows for a holistic understanding of how social work can intervene across the timeline of these women's lives—before, during, and after incarceration. Your⁠ recommendations will naturally draw on this longitudinal perspective to⁠ promote empowerment, dignity, and reintegration through targeted, gender responsive, and holistic interventions.

## 5 Discussion of major findings

This study explored women's lived experiences of incarceration through the lens of psychological empowerment, highlighting their struggles with identity loss, emotional distress, and systemic neglect, as well as⁠ their aspirations for growth and reintegration. Rather than framing the experience simply as “sustaining life” in prison, the findings illuminate how empowerment manifests in intrapersonal confidence, critical awareness of social and institutional barriers, and behavioral intentions toward future advocacy and self-development. The role of social work, though currently limited and underdeveloped, is perceived as potentially vital in fostering these empowerment processes by providing psychosocial support, gender-responsive services, and reintegration preparation⁠. The findings are interpreted through the lens of Empowerment Theory (28), which emphasizes individuals' capacity to gain control over their lives, as well as Feminist Criminology, which foregrounds gendered power dynamics within justice systems (38).

### 5.1 The experience of female prisoners

A key finding was the depth of psychological distress associated with incarceration, particularly due to separation from children and family—an issue more acutely experienced by women than men. This aligns with Feminist Criminology, which highlights how car⁠ceral systems overlook the caregiving roles, social stigma, and trauma histories that uniquely affect women (39, 40).

Participants' descriptions of isolation, loss of identity, and emotional neglect reflect a breakdown in intrapersonal empowerment—one of the three⁠ components in (28) empowerment model. Their capacity for self-efficacy, hope⁠, and personal agency was significantly reduced. Furthermore, the prison's failure to provide meaningful social or emotional support⁠ damaged participants'⁠ interactional empowerment, or belief in their⁠ ability to influence their surroundings.

In addition, the participants' experiences echoed Sykes' (41) concept of the “pains of imprisonment” and Birmingham's (42) work on prison induced mental health strain. Yet, despite these challenges, many women developed internal resilience. Their reflections on learning from adversity indicate the potential for behavioral empowerment—the third element of Zimmerman's framework—even under conditions of extreme constraints.

### 5.2 Experiences with social work practices

The findings indicate a significant disconnect between policy and practice in the delivery of social work services. While some participants mentioned informal “counseling,” none reported receiving structured therapeutic support, vocational⁠ training⁠, or reintegration planning—services mandated by the Ethiopian Federal Prisons Proclamation No.⁠ 365⁠/2003 (43).

This suggests that the current system fails to embody the principles of empowerment-oriented social work, which emphasize enabling choice, building capabilities, and supporting active participation in personal transformation. According to Pollack (44) and Simon (45), when correctional social work focuses only on control or emotional support, it risks reproducing powerlessness instead⁠ of mitigating it.

The lack of formal support also contravenes global best practices. In countries like the UK and India, female-centered rehabilitation programs involve mufti-⁠sectorial collaboration, trauma-informed care, and comprehensive skill-building—elements notably absent in the Ethiopian context. In this way, the Ethiopian prison system demonstrates what critical social work theorists call “⁠structural neglect,” where institutional policies overlook systemic inequities in favor of surface level⁠ compliance.

This is a critical failure from an empowerment theory standpoint, which sees empowerment⁠ not just as personal healing, but as access to social resources, meaningful roles, and life choices. The participants' calls for education, vocational training, and staff development reflect a demand for precisely these empowerment pathways.

### 5.3 Prospects of life after prison

Despite the adversities they faced, many⁠ participants expressed hope for transformation—⁠a testament to their resilience. Several envisioned returning to education, contributing to their communities, or becoming advocates for justice. These expressions of future orientation reflect latent empowerment—the belief that one's life can improve, even in the absence of institutional support.

However, the lack of reintegration programs, job training, and aftercare leaves these women vulnerable to unemployment, recidivism, and social marginalization, as highlighted in prior research (7–9). This gap between aspiration and opportunity illustrates the limitations of empowerment in environments that offer no structural pathways for⁠ actualizing change.

Empowerment Theory stresses that real empowerment requires a combination of internal belief and external opportunity. While participants have demonstrated the former, the correctional system has largely failed to provide⁠ the latter.

In general;

- Incarcerated women face profound psychological distress, particularly due to separation from children and family.
- Prison environments exacerbate mental health challenges, with limited emotional and social support.
- Social work services are narrowly focused on counseling, lacking comprehensive training and reintegration programs.
- There exists a gap between policy and practice in Ethiopian correctional facilities⁠, particularly concerning rehabilitation.
- Despite the shortcomings, female inmates remain hopeful and express a desire for meaningful life changes post-release.

## 6 Conclusion and implications

### 6.1 Conclusion

The lived experiences of incarcerated women in this study reveal the often neglected rehabilitative⁠ aspect of correctional social work. The findings indicate that the primary focus of correctional social work, as it currently stands, is to help women adapt to prison life—yet even this support is widely perceived as inadequate by the women themselves. Participants demonstrated limited awareness⁠ of the services available, further reflecting the disconnect⁠ between institutional goals and practical outcomes.

By evaluating social work practices from the perspective of those directly impacted, this study⁠ has uncovered critical blind spots. Notably, many participants held negative views of the personnel providing social services, highlighting a trust deficit that impedes the delivery and effectiveness of such services. Additionally, the women were largely unaware of what correctional social work entails, underscoring the need for greater outreach, education, and engagement.

The U⁠N Standard Minimum Rules for⁠ the⁠ Treatment of Prisoners assert that prisons should serve reformative, restorative, and rehabilitative functions. A prison sentence should prepare inmates to lead law-abiding, self-supporting lives upon release through access to education, vocational training, and treatment for drug or alcohol dependency. However, research shows that women's⁠ prisons typically offer fewer opportunities in these areas compared to men's facilities (46).

Despite these systemic shortcomings, the study participants remained hopeful about their future and expressed a desire to build better lives after prison. This⁠ optimism is an important asset that the institutions should nurture. The⁠ prison system should capitalize on this potential by providing structured programs that empower women with skills, knowledge, and mental resilience. As Ronald (47) emphasized, correctional institutions⁠ must prepare inmates for successful reintegration, including opportunities to earn money and build self-sufficiency while incarcerated.

### 6.2 Implications

#### 6.2.1 Implications for social work practice

The⁠ findings suggest that⁠ the classification of inmates based on their specific social work intervention needs is essential. Although the participants were all convicted prisoners, their perceptions and attitudes toward social work services mirrored those of individuals⁠ on remand. This implies a universal shortfall in service delivery, irrespective of legal status.

This study effectively serves as a feedback mechanism for prison social work services. It offers valuable insights that could prompt institutional introspection and encourage⁠ the correctional⁠ system to⁠ address gaps and limitations. Social work interventions should be coordinated across⁠ all levels of the correctional system and should aim to achieve social justice through systemic reform.

Furthermore, the study contributes to the limited body of research on incarcerated women from a social work perspective.⁠ Most existing literature focuses on human rights issues, while the unique rehabilitative and psychosocial needs of women in prison remain underexplored. It is hoped that this study will inspire further research and program development aimed at addressing the deficiencies⁠ in correctional social work uncovered here.

#### 6.2.2 Implications for policy makers and NGOs

There is a pressing need for penal reform policies that address gender inequality and the specific vulnerabilities of women. Laws⁠ must be strengthened to improve legal aid and legal awareness for women, particularly in cases of domestic violence—thereby reducing unjust detention. Additionally, greater representation of women in⁠ legislative bodies is necessary to ensure their voices shape policy.

An independent oversight body—⁠comprising NGOs, civil society organizations, and other stakeholders—should be established to monitor prison administration. Such a body would promote transparency, accountability, and continuity of rehabilitation efforts after release. This would ensure that services don't stop at the prison gate but follow women back into society, supporting effective reintegration.

Public–private partnerships should be encouraged in the administration and reform⁠ of the criminal justice system⁠. These partnerships could enable more efficient and humane service delivery. NGOs, in particular, could play a key role by addressing staffing shortages, providing training, and advocating for the rights and needs of incarcerated women.

## Data Availability

The original contributions presented in the study are included in the article/Supplementary Material, further inquiries can be directed to the corresponding author.
